# Effects of Competition, Drought Stress and Photosynthetic Productivity on the Radial Growth of White Spruce in Western Canada

**DOI:** 10.3389/fpls.2017.01915

**Published:** 2017-11-07

**Authors:** Syed A. Alam, Jian-Guo Huang, Kenneth J. Stadt, Philip G. Comeau, Andria Dawson, Guillermo Gea-Izquierdo, Tuomas Aakala, Teemu Hölttä, Timo Vesala, Annikki Mäkelä, Frank Berninger

**Affiliations:** ^1^Key Laboratory of Vegetation Restoration and Management of Degraded Ecosystems, Guangdong Provincial Key Laboratory of Applied Botany, South China Botanical Garden, Chinese Academy of Sciences, Guangzhou, China; ^2^Department of Physics, University of Helsinki, Helsinki, Finland; ^3^Department of Forest Sciences, University of Helsinki, Helsinki, Finland; ^4^Forest Management Branch, Alberta Agriculture and Forestry, Edmonton, AB, Canada; ^5^Department of Renewable Resources, University of Alberta, Edmonton, AB, Canada; ^6^Department of General Education, Mount Royal University, Calgary, AB, Canada; ^7^INIA-CIFOR, Madrid, Spain

**Keywords:** boreal forest, drought, photosynthetic production, tree-tree competition, tree growth, western canada

## Abstract

Understanding the complex interactions of competition, climate warming-induced drought stress, and photosynthetic productivity on the radial growth of trees is central to linking climate change impacts on tree growth, stand structure and in general, forest productivity. Using a mixed modeling approach, a stand-level photosynthetic production model, climate, stand competition and tree-ring data from mixedwood stands in western Canada, we investigated the radial growth response of white spruce [*Picea glauca* (Moench.) Voss] to simulated annual photosynthetic production, simulated drought stress, and tree and stand level competition. The long-term (~80-year) radial growth of white spruce was constrained mostly by competition, as measured by total basal area, with minor effects from drought. There was no relation of competition and drought on tree growth but dominant trees increased their growth more strongly to increases in modeled photosynthetic productivity, indicating asymmetric competition. Our results indicate a co-limitation of drought and climatic factors inhibiting photosynthetic productivity for radial growth of white spruce in western Canada. These results illustrate how a modeling approach can separate the complex factors regulating both multi-decadal average radial growth and interannual radial growth variations of white spruce, and contribute to advance our understanding on sustainable management of mixedwood boreal forests in western Canada.

## Introduction

Boreal forests cover roughly one-third of the global forest area, but, owing to their cold climate and the slow decomposition rate of their coniferous biomass, these ecosystems contain half of the global forest carbon (Jiang et al., 2016). Global warming is projected to continue, regional droughts may intensify and become more frequent during this century, and reduction of carbon uptake by these northern hemisphere high latitude forests is projected (IPCC, 2007; Stephens et al., 2007; Piao et al., 2008; Girardin et al., 2016a). Although Japanese and Finnish boreal regions have reported recent growth enhancements, attributed to elevated CO_2_ and N deposition (Fang et al., 2014; Kauppi et al., 2014), Girardin et al. (2016b) did not find consistent growth responses in the Canadian boreal forest under a half-century of combined warming and CO_2_ fertilization. Since the Canadian boreal makes up 30% of the boreal forest worldwide, it plays a critical role in the global carbon budget (Kurz et al., 2008). The carbon balance of Canadian boreal forests is already affected by climate change through increased fire frequency, unprecedented expansion of insect outbreaks, and widespread drought-induced tree mortality (Ma et al., 2012). Western Canada's boreal forest may become a net carbon source if climate change-induced droughts continue to increase (Ma et al., 2012).

Modeling forest response to climate is complex. Linear models of climate effects on tree growth (e.g., Huang et al., 2010) are effective for identifying useful predictors of growth and the direction of future climate effects; however, since the effects of climate are myriad and complex, linear models may demonstrate poor behavior under future extrapolations of climate. Process-based models can be more effective for this reason, applying better-conditioned models to yield more robust predictions. A common process approach is to analyse tree growth as a function of primary production and allocation to the stem (e.g., Berninger and Nikinmaa, 1997); previous work has shown reliable correlations between photosynthetic production and tree growth (e.g., Berninger et al., 2004; Babst et al., 2014). Gea-Izquierdo et al. (2010, 2014) developed an effective gross primary production model that tracks changes in photosynthetic capacity during the growing season, from the rise in spring temperature to the onset of fall frost. The model was calibrated with data from Canadian carbon flux towers (Gea-Izquierdo et al., 2010), and subsequently tested on long-term tree ring growth data in several regions (Gea-Izquierdo et al., 2014). By capturing the process of climate effects on seasonal photosynthetic capacity, this model effectively adjusts for shifts in the timing of the spring flush, onset of dormancy, and length of the growing season, reducing the predictors required to model tree ring-growth. In this study, we tested this model against western Canadian mixedwood white spruce (*Picea glauca* (Moench.) Voss) ring-growth records. However, since drought was not a major issue in the development of this model, and stands in which it was calibrated were uniform, single species and single-cohort stands, we further investigated the effects of drought and competition on growth.

Tree drought response is also complex. Unsurprisingly, trees exhibit a distinct reduction in radial growth in response to drought conditions (e.g., Gea-Izquierdo et al., 2012; Deslauriers et al., 2014). It appears that abiotic stress factors (including drought), lead to a reduction in the extent of annual ring increment, altered hydraulic properties and chemical composition of the wood, which further sensitize trees to further drought (Lautner, 2013). Balducci et al. (2014) showed lower density wood formation in black spruce seedlings during droughts, reflecting a lower carbon allocation to cell wall formation, resulting in a hydraulic system poorly adapted to drought. Under water-deficit conditions, differentiating xylem cells may not expand fully because of the lack of turgor pressure (Steppe et al., 2015; Deslauriers et al., 2016). The higher sensitivity of growth to drought in comparison to photosynthesis (Hsiao and Acevedo, 1974; McDowell, 2011; Tardieu et al., 2011) also typically leads to an accumulation of non-structural carbohydrates at least in the initial phases of drought (e.g., Mencuccini, 2014; Mitchell et al., 2014). Drought-induced water stress has been demonstrated to be the dominant contributor of widespread tree mortality, and growth decline in the western boreal forests of Canada (Hogg et al., 2008; Ma et al., 2012). In this region, substantial warming and increased drought stress is predicted over the next century (Price et al., 2013; Wang et al., 2014). We employed the Canadian drought code as an effective process-oriented predictor (Turner, 1972; Girardin et al., 2001) to index annual drought stress. This drought index tracks the available rooting zone water profile over the course of the growing season following spring thaw, allowing for evapotranspiration loss and precipitation recharge.

Further complexity is introduced by the mixed-species nature of much of the boreal forest. The western boreal consists of mixtures of trembling aspen (*Populus tremuloides* Michx.), a shade-intolerant species, and white spruce, a shade-tolerant species along with some shade-intolerant pines and several other tree species. These compete for physical space and resources in both an asymmetrical fashion, where taller trees receive disproportionately more light than shorter stems, or in a symmetric fashion, where scarce below-ground resources affect trees in proportion to their size (Weiner, 1990; Schwinning and Weiner, 1998) though there may be differences among species. The effects of competition on tree growth is thus dependent on the number, size and species of neighboring trees (Huang et al., 2013). Shade-tolerant species usually achieve a higher net carbon gain than shade-intolerant species in similarly shaded environments because of their lower respiration rates (Valladares and Niinemets, 2008). Mixedwood stands generally stratify in height by species due to different height growth patterns and shade tolerance characteristics among species (Larson, 1992). Climate change may intensify the effects of competition (Metsaranta and Lieffers, 2008; Luo and Chen, 2015), which makes consideration of competition important in modeling the consequences of climate change.

Many studies have already highlighted how competition interacts with growth responses to climate in different tree species and forest biomes (e.g., Weber et al., 2008; Lebourgeois et al., 2014; Ruiz-Benito et al., 2014; Fernández-de-Uña et al., 2015, 2016; Trouvé et al., 2015). However, we do not know of any published studies that have examined the interactions among drought stress, potential gross photosynthetic productivity (GPP) and competitive status of trees. The main objective of our study is to understand the interactions of drought, simulated stand photosynthetic productivity and competition on the radial growth of white spruce. We hypothesized that modeled GPP, drought and competition determine the radial growth of white spruce trees, and dominant trees are more sensitive to environmental factors (GPP and drought) than suppressed trees.

## Materials and methods

### Study area

**Figure 1:**
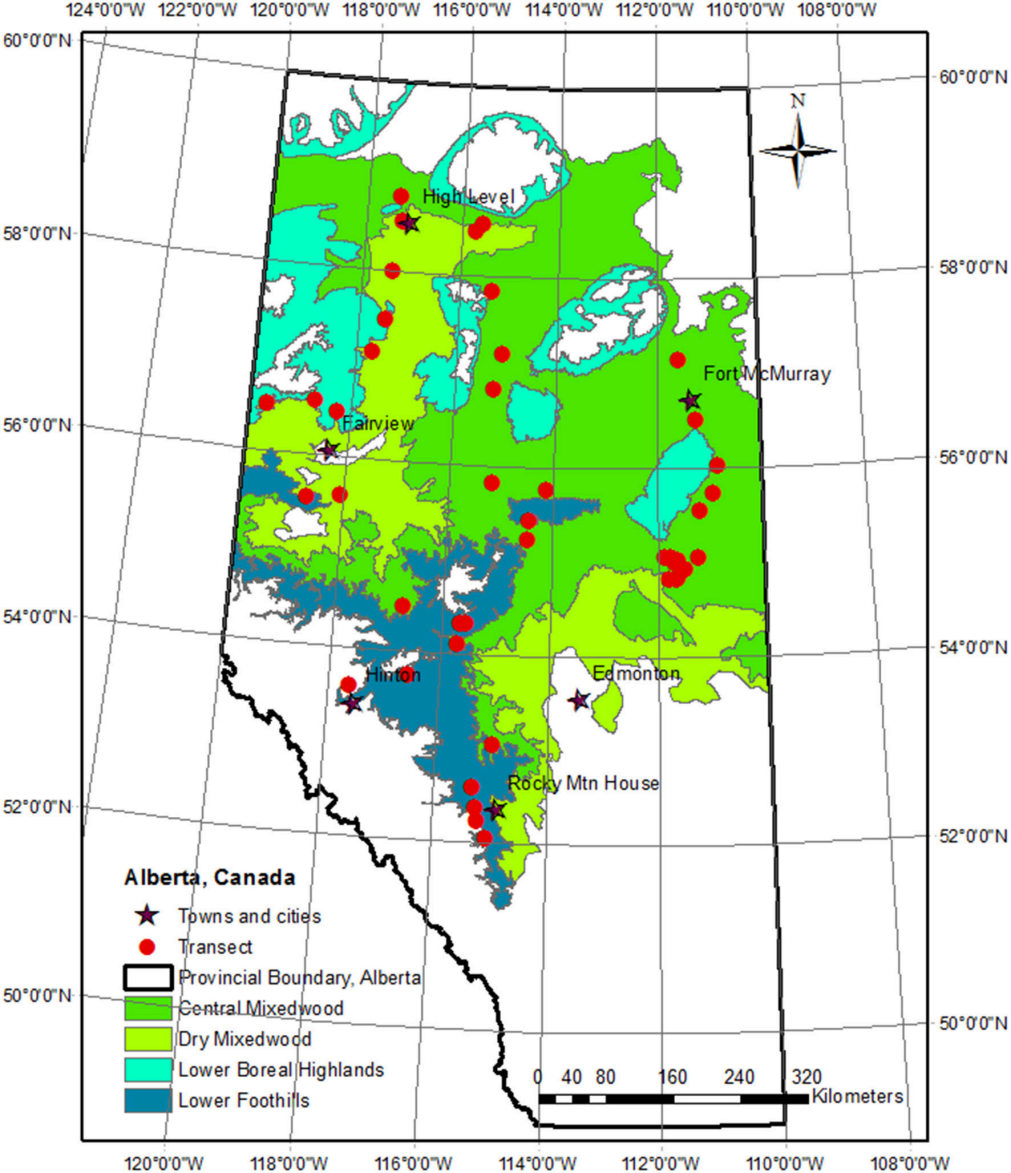
Transect locations within the study region of western Canadian boreal mixedwood forests.

Alberta has some of the most diverse terrain in North America and covers an elevation ranging from 210 m in the northeast to 3,700 m in the western Rocky Mountains. The majority of the forest area in Alberta is contained in the Boreal Plains ecological region. This region has four natural sub-regions (Figure 1): central mixedwood, dry mixedwood, lower boreal highlands, and lower foothills; based on analysis of regional differences in vegetation, soil, site, climate conditions, and forest composition (Beckingham and Archibald, 1996; Beckingham et al., 1996). There is a considerable area of wetlands; however, only the productive upland forests were considered in this study. The main soil types on these upland sites include orthic gray luvisols and brunisols with silty or clay loam texture (Huang et al., 2013). A dry continental boreal climate prevails with warm summers and cold winters. The 1971–2000 climate normals indicate that, in this region, mean annual temperature (MAT), growing degree days (>5°C) and mean annual total precipitation (MAP) were 2.0°C, 1,306°Cd, and 487 mm with 30% in the form of snow, respectively (Huang et al., 2013; Jiang et al., 2016). Throughout these natural sub-regions, MAT decreases from south to north and east to west, while MAP increases with elevation; the driest areas are located in the southeast and in the broad Peace River valley in the northwest (Environment Canada, 2012).

The boreal forest here, and across western Canada, is dominated by intimate mixtures of trembling aspen and white spruce, with lodgepole pine (*Pinus contorta* Douglas ex Loudon) co-occurring at higher elevations in the west and jack pine (*Pinus banksiana*) on sandy soils in the east (Cumming et al., 2000; Stadt et al., 2007; Huang et al., 2013). Lesser amounts of balsam poplar (*Populus balsamifera* L.), paper birch (*Betula papyrifera* Marsh.) and balsam fir (*Abies balsamea* (L.) Mill.) also occur. The aspen-spruce mixedwoods are found on all but the driest and wettest ecosites. Both inter- and intra-specific competition is a significant driver of tree growth and succession in these forests (Stadt et al., 2007; Huang et al., 2013).

We randomly selected 44 accessible mixedwood stands, located between the latitudes of 52 and 59°N, and longitudes of 110 and 120°W (Figure 1). Elevations of the sites ranged from 266 to 1,308 m. Selection was done so that the sites covered a range of latitude and elevation, and were within several km of the major roads. Extreme uneven-aged stands or stands with indications of ground fires were excluded. According to the Phase 3 inventory database (Alberta Forest Service, 1985), stand age ranged from 25 to 100 years at the time of sampling. Sampled stands were distributed throughout the four natural sub-regions.

### Climate data

The climate data used in our study were generated using ANUSPLIN (version 4.3) (Hutchinson, 2004), which uses thin plate smoothing splines to develop elevation-dependent spatially continuous climate surfaces from sparse weather station data (McKenney et al., 2006). Daily climate data from 1930 to 2010 was generated from ANUSPLIN for corresponding latitudes of sampled transects. In this study, the following climate variables were used: daily mean temperature, mean annual maximum temperature, mean annual minimum temperature, temperature sum (TSUM = growing degree-days), mean annual maximum drought code, MAT and MAP. The TSUM is the daily mean temperature above +5°C, summed over the year. Mean annual maximum drought code (hereafter referred as drought code) was calculated from the daily growing season drought code (from May to October), estimated from daily maximum temperature and precipitation data as described by Girardin and Wotton (2009). This Canadian drought code is a cumulative daily rating of the moisture content of typical forest soils calculated from the daily meteorological records or estimates following each spring thaw (Turner, 1972; Terrier et al., 2014). This code has been shown to be a good indicator of the hydric stress of trees (Girardin et al., 2001). The minimum drought code value of zero represents a fully recharged soil rooting zone, a value of 200 is an indication of high drought severity, and 300 or more is extreme (Girardin et al., 2004; Terrier et al., 2014). Since we needed an annual index to match the annual resolution of the tree rings, we chose the maximum value of the drought code which occurred over each growing season. Over the study region during the 80 year study period (1930–2010), MAT ranged between −2.27 and 3.22°C, MAP varied from 263.9 to 633.3 mm, and maximum annual drought code from 221.4 to 672.3. Therefore, it is evident that our study area routinely experiences high to extreme droughts.

### Tree ring and inventory data

Field sampling was conducted from 2007 to 2011. A belt transect as used by Huang et al. (2013) was employed, 5–80 m long, and 5–20 m wide. Transect area ranged from 25 to 1,600 m^2^ (mean = 484m^2^); the length and width were varied to distribute sampling over each stand's area and capture sufficient spruce. From each transect, 10 to 20 live white spruce were randomly chosen as subject trees to be felled for tree ring analysis. These spruce ranged from suppressed trees to co-dominant. Height, DBH (diameter at a breast height of 1.3 m) and a competition assessment were taken before felling, then a stem disk taken at 0.3 m height for tree ring analysis. In the same stand, five of the thickest DBH (typically codominant) white spruce trees were also selected to measure potential growth on the site. The DBH of these largest spruce trees were measured and two 5.1 mm increment cores per tree were collected at 1.3 m height. The conversion of DSH (diameter at stump height of 0.3 m) into DBH was done following the equations developed by Huang et al. (2013) and therefore, we assumed that the ring width (= radial growth) taken at stump height behaves similarly to ring width taken at 1.3 m height (dominant trees). In total, 852 spruce trees (632 subject trees plus 220 largest trees) were sampled from 44 transects for stem growth and competition-level assessment.

All tree-ring samples (cores and discs) were dried and polished with fine grits of sandpaper. From each disc, two radii were chosen for measurement, separated by an angle of 90–180°, avoiding knots and severe reaction wood. All tree ring samples (cores and radii) were carefully measured using a stage micrometer measuring system interfaced with the “Time Series Analysis Program” (TSAP; Frank Rinntech, Heidelberg, Germany) to a precision level of 0.001 mm. Visual cross-dating was verified using COFECHA (Holmes, 1983). The correlation between individual series and the master chronology within each transect was well above 0.55 and significant at *P* < 0.01, which indicates strong similarities in inter-annual ring growth pattern among trees within transects. Master chronologies between nearby transects were also well correlated (*r* > 0.48, *P* < 0.01) indicating the cross-dating was reliable. We used regional curve standardization (RCS; Esper et al., 2003) for detrending individual tree ring-width series to remove age-dependent growth trends. Tree ring indices were calculated as the residuals of the predicted and measured growth.

### Competition index

For competitor assessment, the DBH of all trees and shrubs taller than 1.3 m and within a 1.78 m radius plot (0.001 ha) centered at the subject tree were considered. Since each subject tree was randomly selected in each transect, we used these small assessment plots as samples of transect level structure. We combined all competitors from all subject trees, including all subject trees except the current one and then calculated stand basal area [ = π × Σ (DBH/2000)^2^/(0.001 ha × number of competition plots); DBH in mm]. To assess the stand level growth-competition relationship of white spruce, two competition indices, namely stand basal area (m^2^ ha^−1^) and relative height were used. Basal area was grouped into three functional classes by summing up the basal area per hectare of deciduous trees (trembling aspen, white birch and balsam poplar), of pines (lodgepole pine or jack pine) and of white spruce. The relative height of each subject spruce was calculated by dividing subject tree height by the maximum height of sampled spruce within each transect. Competition was necessarily current competition (stand basal area and relative height at the time of sampling) and we assumed that current competition is an indicator of past competition. Unfortunately, due to the rapid decomposition of the deciduous component of these stands, we cannot reconstruct past competition. Even though the relative variation of productivity is not same for dominant and suppressed trees because of the shift in the mode of competition (symmetric-asymmetric), a similar behavior in productivity is expected for them.

### The photosynthetic production model

In a previous study, net ecosystem exchange data at 12 evergreen coniferous forests from northern temperate and boreal regions was used by Gea-Izquierdo et al. (2010) to fit a photosynthesis production model. In our study, we used the model parameterization presented in Gea-Izquierdo et al. (2010, 2014) to simulate daily stand GPP at each site and analyse its relationship with radial growth variability. The daily GPP per unit ground area (*A(t)*, mol m^−2^ day^−1^) was modeled for each of the 44 transects by using daily climate data from our study sites. Within the model, *A(t)* is a nonlinear function of stomatal conductance of carbon dioxide (*g(t)*, mol CO_2_ m^−2^ day^−1^), photosynthetic capacity (α*(t)*, mol CO_2_ m^−2^ day^−1^), and a saturating function of average light intensity (γ*(t)*, dimensionless):

$$\begin{array}{l} A\left(t\right)=\;\frac{g\left(t\right){\;C}_{a\;}\alpha \left(t\right)\;\gamma \left(t\right)}{g\left(t\right)+\alpha \left(t\right)\;\gamma \left(t\right)} \end{array}$$

where the stomatal conductance is expressed as

$$\begin{array}{l} g\left(t\right)=\text{max}\left(0.00001,\;\tilde{g}\left(t\right)\right),\text{with}\\ \tilde{g}\left(t\right)=\left(\sqrt{\frac{C_{a}\;10^{-6}\;\lambda }{1.6\;D\left(t\right)}}-1\right)\alpha \left(t\right)\;\gamma \left(t\right) \end{array}$$

and the light response of biochemical reactions of photosynthesis:

$$\begin{array}{l} \gamma \left(t\right)=\;\frac{Q\left(t\right)}{Q\left(t\right)+\delta } \end{array}$$

where,

*C*_*a*_ is the air CO_2_ concentration in ppm, varied over 80 years (1930–2010) e.g., linearly from 330 to 400 ppm,

*Q(t)* is the average daily incident photosynthetically active radiation (μmol m^−2^ s^−1^),

*D(t)* is the water vapor pressure deficit (kPa) calculated using maximum daily temperature estimates above the tree canopies,

δ is the half saturation parameter of the light function (μmol m^−2^ s^−1^), and

λ is a model parameter, set here to 3,000 kPa (Gea-Izquierdo et al., 2010), expressing the sensitivity of stomatal conductance to water vapor pressure deficit,

Photosynthetic capacity, α*(t)* was modeled as a lagged function of temperature *S(t)*, following Gea-Izquierdo et al. (2010):

With

$$\begin{array}{l} \alpha \left(t\right)=\;\alpha_{max}/\left(1+\exp\left(b\;\left(S\left(t\right)-\;T_{s}\right)\right)\right), \end{array}$$

and *S(t)* from

$$\begin{array}{l} \frac{dS\left(t\right)}{dt}=\;\frac{T_{air}\left(t\right)-S\left(t\right)}{\tau } \end{array}$$

*T*_*air*_ *(t)* is the measured mean daily air temperature (°C) at time *t*,

α_*max*_ (mol m^−2^ day^−1^) is the maximum photosynthetic efficiency, which takes into account whole canopy properties,

*b* (°C^−1^) is the curvature of the sigmoid function,

*T*_*s*_ (°C) is the inflection point of the sigmoid curve, i.e., the temperature at which α reaches half of α_*max*_, and τ (days) is the time constant of photosynthetic acclimation and indicates the time it takes for photosynthetic capacity to acclimate itself to changing temperature.

The production model was run for each growing season to generate an integrated annual estimate of GPP for the year to compare with the annual tree ring data.

### Modeling approach

The modeling was done in two stages. First we identified the “best” model from an average chronology of all trees at all sites (model identification). Thereafter, we applied this model to all trees in all stands individually in a second stage (which we call model estimation). Model identification followed usual procedures for time series modeling following the stages described by Box et al. (2015). We tested both spline- and RCS-detrended chronologies for the analyses, but decided to abandon the spline-detrending because it yielded consistently poorer results than the RCS. During model identification we used both untransformed and differentiated values of independent variables at different time lags to estimate the cross correlation function (CCF). For final reporting, we retained CCF using untransformed independent variables. Autocorrelation functions (ACF) were estimated using the Extended Sample Autocorrelation Function (ESACF) criteria. All analyses were done using the R-stat package (R Core Team, 2014). Model estimation was done using linear mixed models using the independent variables and time lags from the model obtained from the identification phase. The annual individual tree growth for the period of 1930–2010 as a function of GPP and drought code were estimated using mixed models in the NLME package of R (Pinheiro et al., 2015) using the restricted maximum likelihood (REML) criteria. The final model had the form of:

$$\begin{array}{l} I_{ijk}=\left(b_{1}+\;\beta_{1i}+\beta_{1ij}\right)+\left(b_{2}+\beta_{2i}+\beta_{2ij}\right)A_{ik}\\ +\;\left(b_{3}+\beta_{3i}+\beta_{3ij}\right)D_{ik}+\;\varphi_{ij}I_{ij\left(k-1\right)}+\;\epsilon_{ijk} \end{array}$$

Where,

*I* is the tree ring index of tree *j* for the site *i* in the year *k*.

*b*_1_, *b*_2_, and *b*_3_ are the fixed intercepts and slopes of the model.

β_1*i*_, β_2*i*_, and β_3*i*_ are the random effects for the site *i*.

β_1*ij*_, β_2*ij*_, and β_3*ij*_ are the random effects for the site *i* and tree *j*, respectively.

*D* is the drought index for the site *i* in the year *k*.

*A* is the annual integral of photosynthetic productivity for the site *i* in the year *k*.

φ is the autoregressive coefficient and ϵ is the error term of the model.

Autoregression of parameter values were estimated using the variance and co-variance structure of the model as described in Pinheiro and Bates (2006). Random effects were assumed to be taken from a normal distribution with a mean of 0 and an estimated standard deviation. Standard deviations for the different random effects were assumed to be uncorrelated with each other and across levels. We chose to analyse effects of average climate and competition *post-hoc*. In other words we extracted the random effects from the mixed model for all sites and for individual trees within sites. The random effects were then regressed on climate and competition variables for all sites and trees.

## Results

**Figure 2:**
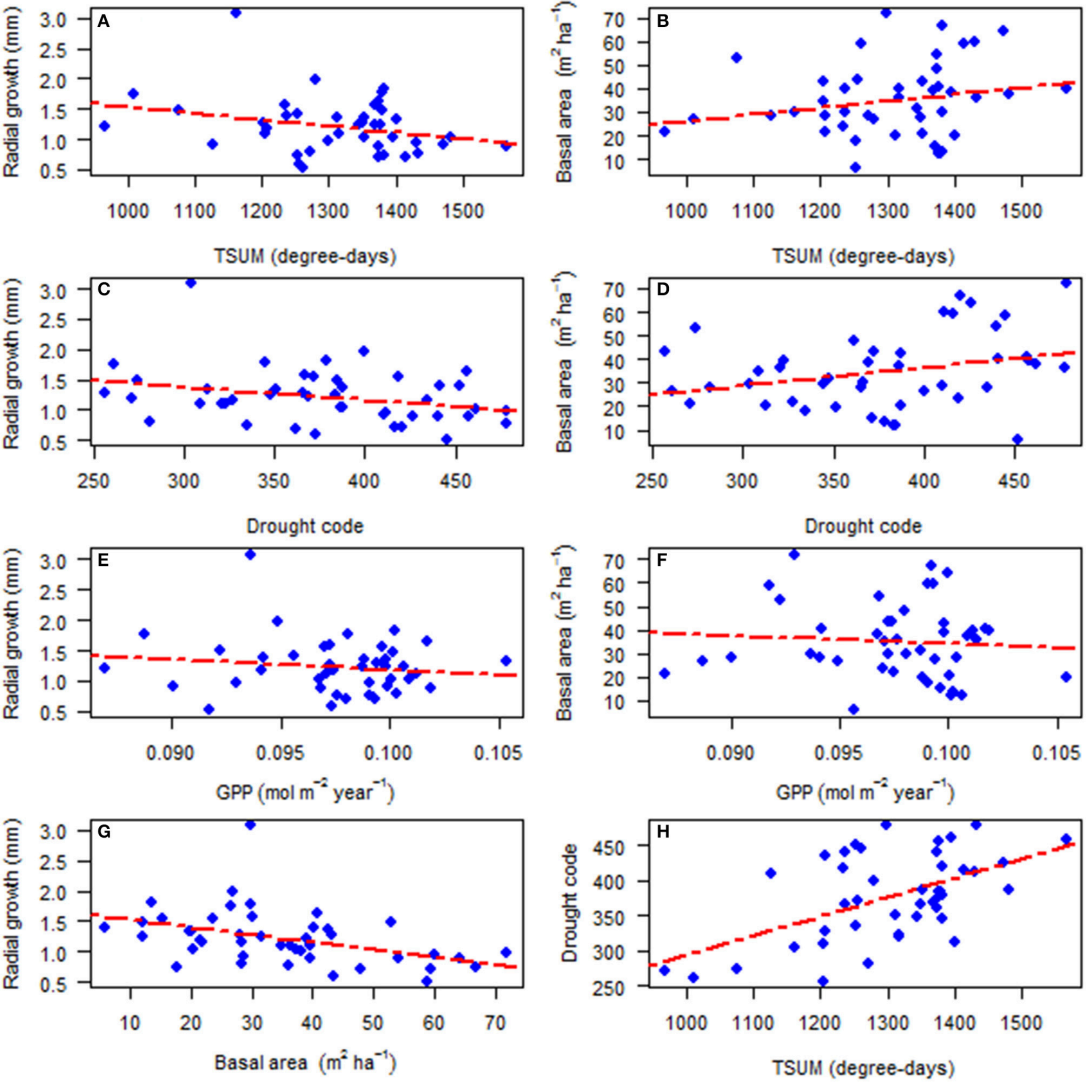
The relationship for the period of 1930–2010 between: **(A)** average radial growth and temperature sum, TSUM (*p* = 0.062); **(B)** transect basal area and temperature sum, TSUM (*p* = 0.174); **(C)** average radial growth and drought code (*p* = 0.058); **(D)** transect basal area and drought code (*p* = 0.054); **(E)** average radial growth and gross photosynthetic productivity, GPP (*p* = 0.360); **(F)** transect basal area and gross photosynthetic productivity, GPP (*p* = 0.608); **(G)** average radial growth and transect basal area (*p* = 0.004); and **(H)** drought code and temperature sum, TSUM (*p* = 0.0001).

Transect characteristics are provided in Table 1, which include location and transect size, diameter and height range of the subject trees, and current basal area of deciduous, pine, spruce and total competitors. The average radial growth of all sampled trees in a stand over their life decreased with the increase of TSUM (Figure 2A; Pearson's correlation: *r*_*P*_ = −0.283, *p* = 0.062) while transect basal area (m^2^ ha^−1^) increased with the increase of TSUM (Figure 2B; *r*_*P*_ = 0.209, *p* = 0.174), but in both cases the relationships were non-significant at 5%. With an increase in the drought code, there was a decrease in average radial growth (Figure 2C; *r*_*P*_ = −0.288, *p* = 0.058) and an increase in transect basal area (Figure 2D; *r*_*P*_ = 0.293, *p* = 0.054) but these relationships were not quite significant. With the increase of modeled GPP (mol m^−2^ year^−1^), both average radial growth (Figure 2E; *r*_*P*_ = −0.141, *p* = 0.360) and transect basal area (Figure 2F; *r*_*P*_ = −0.079, *p* = 0.608) decreased non-significantly. The average radial growth and transect basal area were negatively and significantly correlated with each other (Figure 2G; *r*_*P*_ = −0.422, *p* = 0.004) whereas drought code and TSUM showed a positive and significant correlation (Figure 2H; *r*_*P*_ = 0.539, *p* = 0.0001) with each other.

**Table 1:** Transect characteristics.

**Transect ID**	**Lat., °N**	**Long., °W**	**Transect area, m^2^**	**No. of Subject trees**	**DBH (min), mm**	**DBH (max), mm**	**DBH, (mean), mm**	**Height (min), m**	**Height (max), m**	**Height, (mean) m**	**DECBA, m^2^ ha^−1^**	**PINBA, m^2^ ha^−1^**	**SPRBA, m^2^ ha^−1^**	**Transect basal area, m^2^ ha^−1^**
6	54.83	−111.71	656	18	2.0	175.0	61.2	1.3	14.2	5.7	16.8	0.0	3.6	20.5
7	54.84	−111.68	376	14	14.0	217.0	143.1	1.8	19.3	12.1	32.3	0.0	10.4	42.7
8	54.93	−111.54	1180	13	39.0	199.0	128.1	2.8	16.0	10.4	23.3	0.0	4.6	27.9
9	54.83	−111.84	900	14	21.0	203.0	134.5	2.3	15.7	10.9	12.1	0.0	0.0	12.1
10	55.00	−111.73	810	14	44.0	235.0	153.7	4.5	23.2	14.0	23.2	0.0	15.8	39.0
A	55.03	−111.68	544	11	42.0	246.0	154.1	4.1	21.5	13.1	13.4	0.0	1.8	15.2
AA	55.73	−110.98	62	15	42.0	240.0	89.1	4.8	13.8	9.4	30.6	0.0	17.4	47.9
B	55.05	−111.27	1500	13	64.0	211.0	139.1	6.8	23.0	12.7	31.7	0.0	0.0	31.7
C	55.22	−114.52	800	20	5.0	348.0	193.8	1.6	22.1	16.8	1.9	0.0	28.1	30.0
CC	55.82	−115.21	143	14	45.0	215.0	68.2	4.6	9.8	7.6	28.5	0.0	14.8	43.4
D	55.76	−114.18	1200	16	18.0	221.0	157.3	2.6	20.5	14.7	16.1	0.0	3.6	19.7
DD	56.80	−115.26	60	14	31.0	320.0	106.2	5.6	14.9	11.9	44.0	0.0	23.0	66.9
E	55.44	−114.50	1600	15	7.0	197.0	171.5	1.6	15.6	12.1	5.5	0.0	24.3	29.8
EE	57.19	−115.11	113	14	40.0	295.0	102.0	5.2	17.1	11.6	26.0	7.5	25.8	59.3
F	55.07	−111.81	1180	12	8.0	263.0	157.3	1.4	23.3	13.2	13.4	0.0	0.0	13.4
G	55.56	−111.24	890	12	26.0	250.0	171.5	2.7	19.9	14.2	11.8	0.0	17.9	29.8
H	55.06	−111.90	840	12	34.0	208.0	129.0	3.3	17.3	11.4	6.0	0.0	6.1	12.1
HH	58.53	−117.29	95	14	84.0	210.0	124.1	10.6	16.8	13.2	49.9	0.0	22.1	72.0
II	58.47	−115.79	180	16	39.0	270.0	95.9	4.5	13.8	9.9	19.6	0.0	16.4	36.1
JJ	57.85	−115.38	90	16	64.0	165.0	91.5	7.1	13.9	11.0	46.0	0.0	18.2	64.2
KK	57.99	−117.42	190	16	53.0	145.0	94.4	8.3	14.0	11.1	29.3	0.0	24.8	54.1
L	53.59	−117.67	400	13	4.0	180.0	58.3	1.4	9.7	4.0	5.8	4.1	11.5	21.4
MM	53.75	−116.62	87	14	46.0	422.0	91.2	5.2	14.8	9.7	10.8	0.9	10.2	21.8
PP	58.78	−117.38	25	15	30.0	212.0	77.1	4.3	11.3	8.9	35.2	0.0	23.6	58.8
Q	54.10	−115.75	1261	16	52.0	364.0	100.2	5.0	14.3	8.3	9.9	0.0	29.8	39.7
QQ	58.54	−115.62	90	18	24.0	245.0	99.4	2.7	14.3	10.8	16.5	0.0	21.7	38.2
R	56.57	−118.75	76	14	39.0	215.0	126.7	8.0	19.0	14.0	13.3	0.0	15.3	28.6
RR	57.48	−117.50	86	16	23.0	206.0	90.1	3.4	12.9	7.7	22.9	0.0	3.8	26.7
S	56.49	−119.69	300	10	39.0	77.0	63.8	4.3	8.0	6.1	4.2	0.0	1.8	6.0
SS	57.14	−117.73	103	15	39.0	335.0	88.6	4.4	8.8	7.4	22.1	0.0	18.1	40.2
T	55.54	−118.74	1200	15	16.0	231.0	136.7	2.1	15.2	10.2	23.5	0.0	0.1	23.6
TT	52.58	−115.35	86	10	29.0	65.0	49.0	3.5	5.3	4.6	17.5	3.7	7.1	28.3
U	56.47	−118.31	120	14	36.0	184.0	156.9	8.8	15.1	13.1	5.6	0.0	22.7	28.2
UU	52.22	−115.25	190	15	33.0	310.0	91.4	3.3	11.6	6.8	19.2	1.4	6.1	26.7
V	55.60	−118.11	1300	14	30.0	201.0	125.8	2.8	17.4	11.9	22.0	0.0	18.9	40.9
VV	52.37	−115.30	229	15	40.0	326.0	85.2	3.2	9.9	6.4	26.3	0.0	16.9	43.2
W	54.32	−115.59	138	13	26.0	74.0	51.9	2.7	14.3	5.1	12.1	0.0	5.7	17.8
WW	52.05	−115.08	76	15	42.0	255.0	106.3	4.3	13.4	8.6	26.8	0.0	26.0	52.8
X	54.32	−115.70	600	14	64.0	251.0	123.4	4.8	19.4	9.6	29.7	0.0	5.1	34.9
XX	56.52	−111.30	153	15	58.0	180.0	86.8	5.4	11.5	8.9	45.2	0.0	14.7	59.9
Y	54.48	−116.79	600	14	41.0	193.0	121.1	2.3	16.6	10.3	29.9	0.0	6.3	36.2
YY	57.15	−111.64	63	15	35.0	221.0	81.7	5.4	12.7	9.4	23.0	0.0	16.6	39.7
Z	53.04	−115.01	600	14	14.0	195.0	101.8	1.7	14.7	9.6	5.8	0.0	14.3	20.1
ZZ	56.03	−110.88	112	15	54.0	238.0	123.8	6.5	15.6	11.8	17.4	0.0	19.8	37.2

*DBH (diameter at breast height, 1.3 m, of the subject tree), Height of the subject tree, DECBA (basal area for deciduous species: trembling aspen, white birch and balsam poplar), PINBA (basal area for lodgepole pine and jack pine) and SPRBA (basal area for white spruce) are transect mean values. Transect basal area is the sum of DECBA, PINBA and SPRBA*.

**Figure 3:**
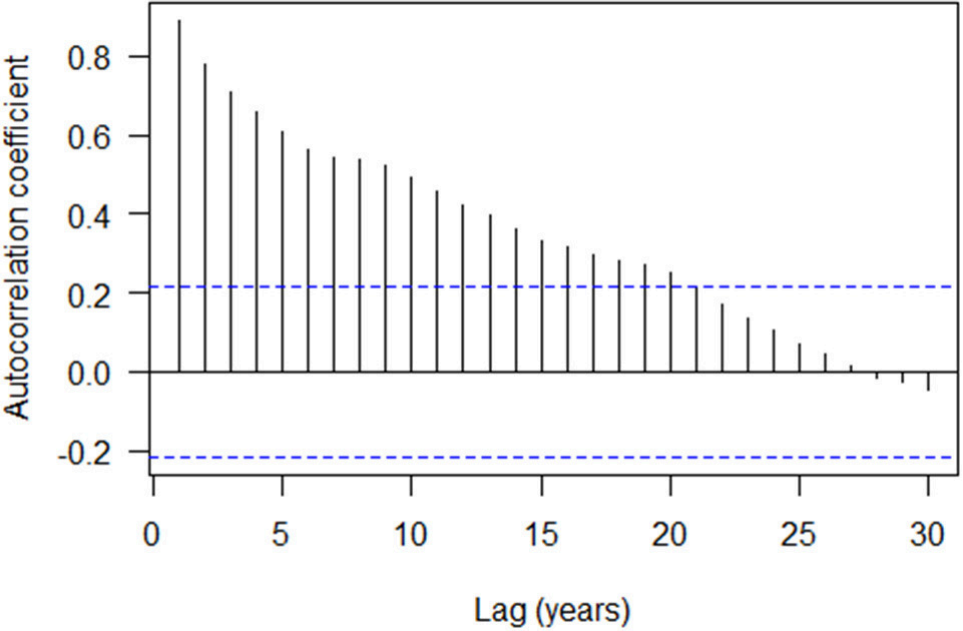
Autocorrelation structure of the tree ring indices (overall mean chronology) with 95% confidence interval. Dotted line is simple approximate confidence interval at $\pm 2/\sqrt{N}$, where *N* is number of years.

**Figure 4:**
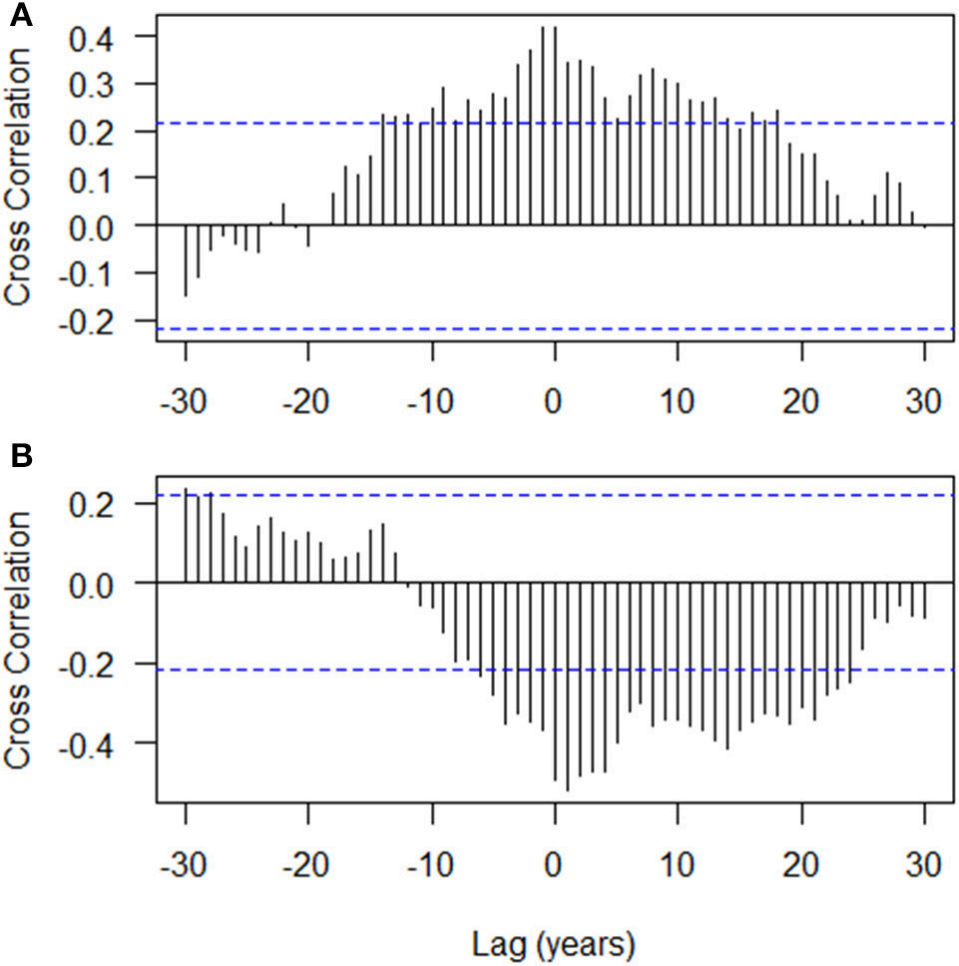
Cross correlation functions of **(A)** tree ring indices (overall mean chronology) with the gross photosynthetic productivity, GPP (mol m^−2^ year^−1^); and **(B)** tree ring indices (overall mean chronology) with the drought code. Dotted line is simple approximate (95%) confidence interval at $\pm 2/\sqrt{N}$, where *N* is number of years.

The autocorrelation structure of the tree ring indices showed that there is strong autocorrelation at lag 1 (0.892) with a gradual decreasing trend over subsequent lags (Figure 3). The ESACF criteria suggested initially that a second order autoregressive model (AR2) would be optimal, however, the 2 years lag was not statistically significant and was subsequently dropped from the model (not shown). The cross correlation of the tree ring indices with the gross photosynthetic production rate showed a moderate positive correlation with no lag (0.420; Figure 4A) while that of the tree ring indices with the drought code revealed a moderate negative correlation at Lag 0 (−0.519; Figure 4B). We used transfer function models for the mean tree and the mean environmental drivers (GPP and drought code) and these indicated that the response of tree ring indices to variation in the environmental drivers is best modeled with no lag (data not shown).

**Figure 5:**
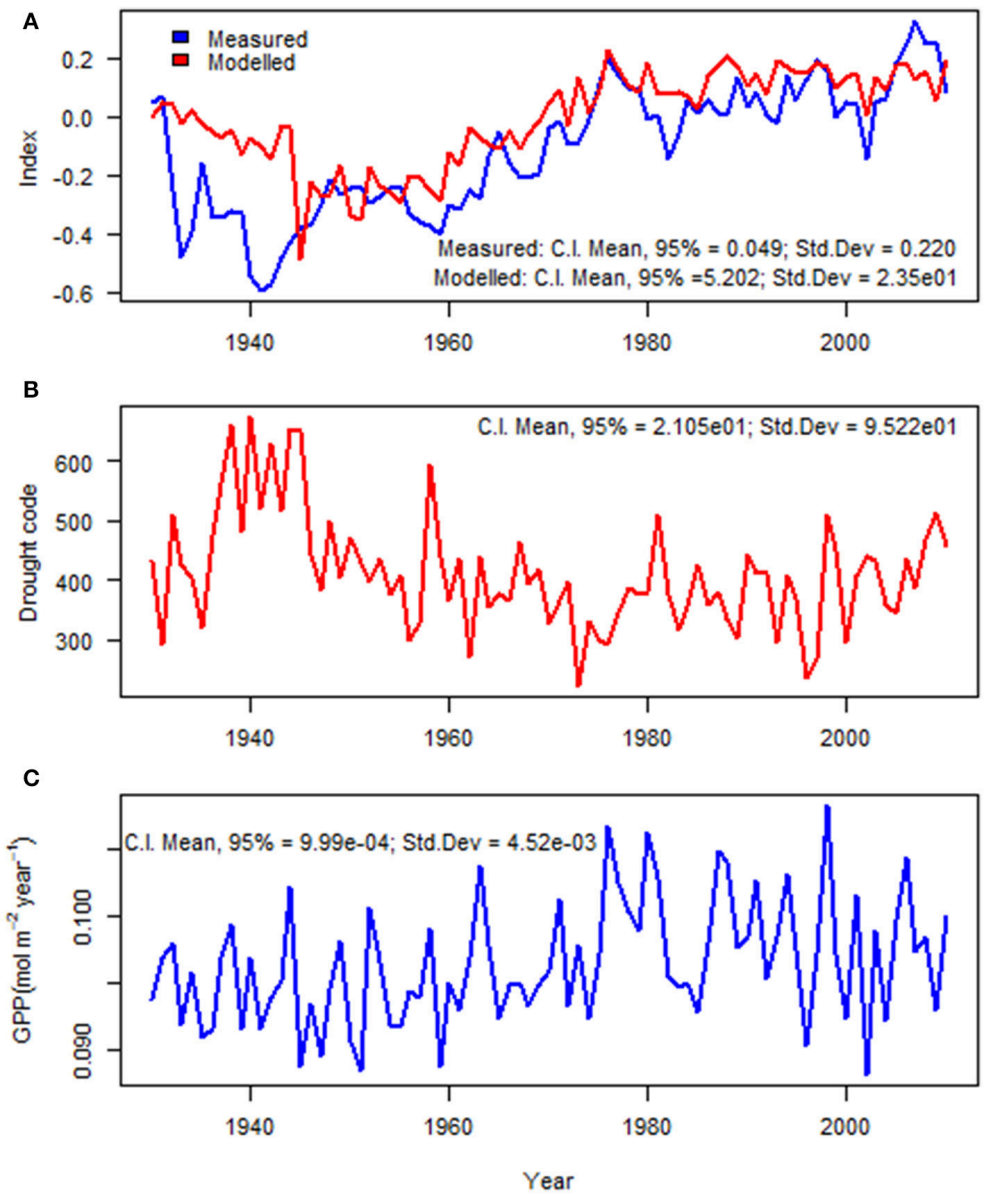
Interannual variations (1930–2010) of **(A)** measured and modeled tree ring indices (model as from Table 2); **(B)** drought code; and **(C)** gross photosynthetic productivity, GPP (mol m^−2^ year^−1^).

The summary of mixed-effects model fit by REML (Table 2) shows that the fixed effects had relatively small errors compared to their means (indicating a statistically significant relationship) and the model had a lower AIC than simpler models. The autocorrelation of the residuals for tree ring growth (AR1) was large (AR1 = 0.83), as also shown in the ACF (Figure 3). The random effects had large variations compared to the mean fixed effects, indicating a large proportion of site specific variation in the regions. The model described well the variation in tree growth except for the period of 1930–1939 (Figure 5A). Severe drought events were apparent in the 1930s (Figure 5B). The time series of GPP gradually increased with time (Figure 5C).

**Table 2:** Summary of mixed-effects model fit.

**Linear mixed-effects model fit**	**Parameter (covariate)**	**Value**	**Std. Error**	**Std. Dev**	***P*-value**
AIC		14,605.18			
AR1		0.826588			
Fixed effects	Intercept	−0.316281	0.088460		0.0004
	Drought index	−0.000153	0.000044		0.0006
	Photosynthetic production	4.954676	0.802193		0.0000
Tree-level random effects	Intercept			0.103754	
	Drought index			0.000002	
	Photosynthetic production			3.728128	
Site (transect)-level random effects	Intercept			0.517795	
	Drought index			0.000260	
	Photosynthetic production			4.414104	

*AIC, Akaike's An Information Criterion; AR1, First order autoregressive term*.

**Figure 6:**
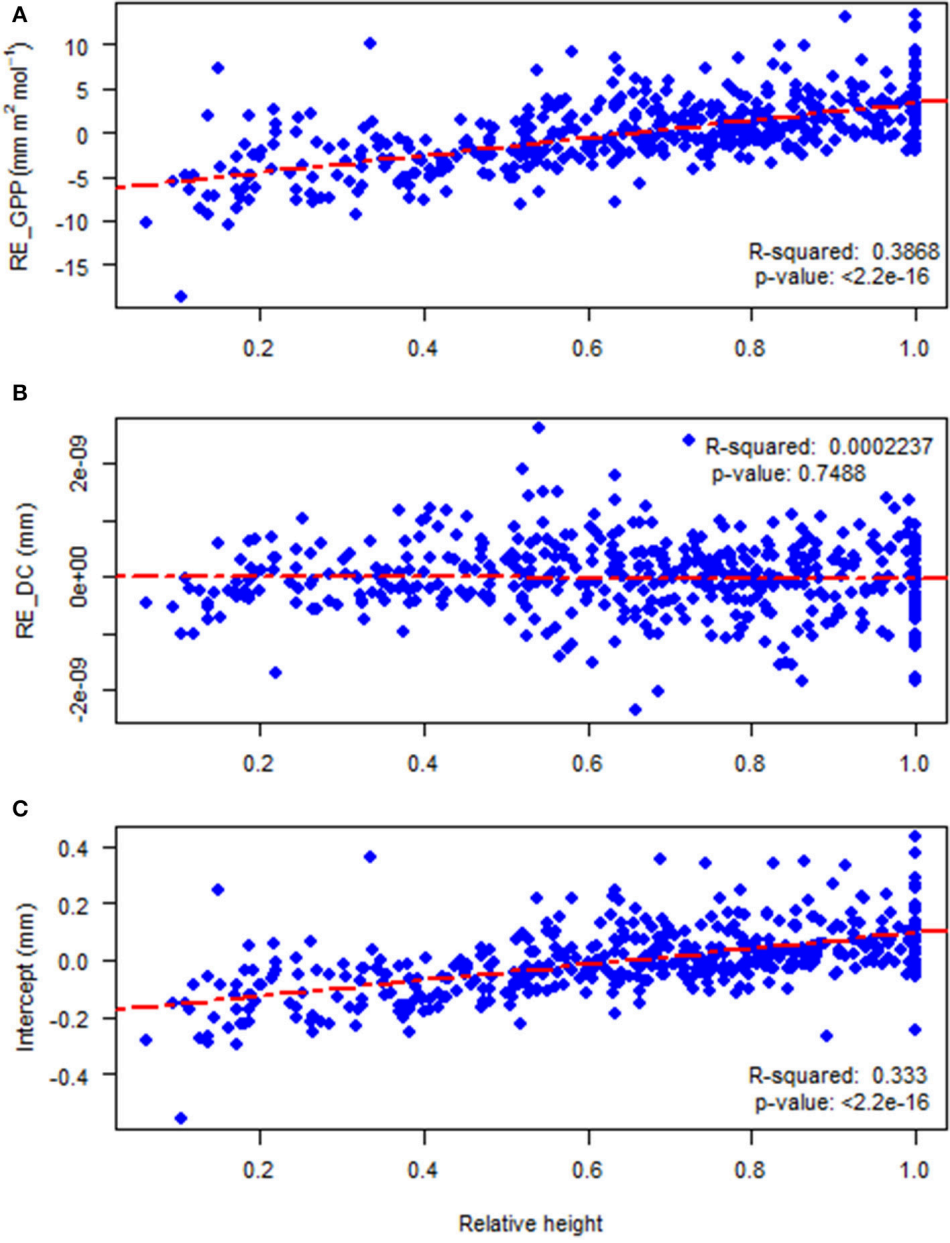
The relationships between: **(A)** tree level random effects of gross photosynthetic productivity (RE_GPP, β_2ij)_ and relative height; **(B)** random effects of drought code (RE_DC, β_3*ij*_) and relative height; and **(C)** tree level random effects on the intercept (β_1ij_) and relative height.

Random effects at the site level did not show any clear geographic pattern or dependence on climate (data not shown). The tree level random effects of photosynthetic productivity (RE_GPP, mm m^2^ mol^−1^, β_2ij_) and the intercept (β_1ij_) of the autoregressive equation demonstrated strongly significant and positive linear correlations with the relative height of subject trees (Figures 6A,**C**; *r*_*P*_ = 0.556, *p* = < 2.2e^−16^ and *r*_*P*_ = 0.498, *p* = < 2.2e^−16^, respectively). The random effects of drought code (RE_DC, β_3ij_) had a non-significant negative correlation (Figure 6B; *r*_*P*_ = −0.078, *p* = 0.093) with the relative height of subject trees.

## Discussion

Tree growth is affected by the processes that operate at different temporal and spatial scales, which is well-recognized in the methods employed for tree ring research. So-called low frequency variations of tree growth are caused by changes in tree age or changes in competition while high frequency variations are caused by climatic variations. Our approach here is similar to the work of Lapointe-Garant et al. (2010) who used a growth model to analyse separately the average growth conditions and climatic variations on tree growth. In our analysis, we tried to contrast which factors affect average radial growth over 80 years (multi-decadal variation) and which factors affect interannual radial growth variations. We used tree-level average ring width for assessing multi-decadal growth variations, and for interannual variations, annual tree ring indices were used.

The modeling approach that we used in this study is a modification of Gea-Izquierdo et al. (2014), who depicted relationship between tree ring width at different temporal scales as a function of photosynthetic production, as well as Berninger et al. (2000, 2004), where the relationship between leaf-level photosynthesis and growth of Scots pine was presented. In our modeling approach, as with Berninger et al. (2000, 2004) and Gea-Izquierdo et al. (2010), we focused on the estimation of climatic effects on stand level photosynthesis and assumed that canopy characteristics are fixed. Drought significantly affects forest productivity, increases vulnerability to biotic disturbances and thereby increases subsequent mortality (Merlin et al., 2015). Therefore, we added a drought index to the model since, in general, radial growth is negatively affected by drought (Corcuera et al., 2004; Drew et al., 2008).

For long-term average tree growth, the role of competition, other stresses and regional climate on the productivity is a matter of ongoing discussion (Kunstler et al., 2011; Bell et al., 2014; Prior and Bowman, 2014; Trouvé et al., 2015; Fernández-de-Uña et al., 2016). Trials to bridge tree rings of individual trees and long-term stand level basal area growth data from forest inventory have been attempted as one of the ways to improve our understanding on how tree growth is regulated at the stand and individual tree scale (Biondi, 1999; Rohner et al., 2016). Our results indicated quite a complex regulation of the long-term radial growth across the sites, and the competition factor regulating the long term average growth of trees differed from the larger suite of factors that regulated year to year variations in growth.

The site-average radial growth of white spruce for the past 80 years showed a non-significant negative relation with the average TSUM (Figure 2A). It seems from this that the growth of white spruce is unlikely to see much benefit under the predicted warming. Goldblum and Rigg (2005) similarly reported that the growth of white spruce may change little under the predicted warming and altered precipitation regime at the deciduous-boreal forest ecotone in Canada. Productivity, measured as simulated GPP, did not have a statistically significant effect on the average stand radial growth (Figure 2E). In other words, the radial growth of individual trees does not increase with increasing GPP while the stand basal area growth might increase since there are more trees with larger basal areas. While there was a significant negative relationship between competitor total basal area and radial growth (Figure 2G), drought code only showed a weak negative relation with radial growth (Figure 2C). Therefore, our results indicate that competition is more important to radial growth than simulated GPP and drought stress. The relationships of radial growth with productivity and drought code are, in a way, similar to the results found by Lapointe-Garant et al. (2010) and Gea-Izquierdo et al. (2014), where they showed marked differences in the responses of trees across different spatial scales.

What makes our data more complex is that there is a positive correlation between stand (transect) basal area and drought index (Figure 2D), and it seems that drier sites encompass a higher basal area. This may be due to the fact that water limitation in our northern ecosystem is not very severe. In a study of terrestrial biomes, for the period of 1981–2006, Vicente-Serrano et al. (2013) indicated that vegetation biomass in northern ecosystems correlates less with drought than southern ecosystems. However, the positive effect of drought index on stand basal area in our study appears to run contrary to the findings of Peng et al. (2011), where they indicated the water stress caused by regional drought may be the dominant contributor to widespread increases in tree mortality across tree species, sizes, elevations, longitudes and latitudes, and thereby reduced growth of forest stands. While stand basal area of our study increased with the increase of TSUM (Figure 2B), the drought code and TSUM correlate strongly and positively with each other (Figure 2H). Consequently, drought code has a similar effect as TSUM on basal area. A comparison study by Ruiz-Benito et al. (2014) across boreal, temperate and Mediterranean biomes in European forests reported that recent climate warming caused an increase in stand basal area but this increase was offset by water availability. Therefore, based on our results, we can infer that a determinant of stand basal area is the heat sum available for tree growth.

The apparent conflict of the results presented in Figure 2 and Table 2, especially the different relationships between GPP and radial growth, can be explained by several factors. In Figure 2, the stand average radial growth is compared to average climate and the final characteristics of the stand. In our data, the stands that have a higher GPP tend to have higher values of drought code and also very different values of basal area. These effects are stronger than the effects of mean GPP on mean growth. This probably causes the non-significant negative relationship between GPP and average radial growth. On the other hand, in Table 2, we presented the effects of interannual variation in GPP to interannual variation in radial growth. Stand level differences in growth are largely absorbed in the variation of the intercept of the mixed model (where there is a large variation in the random intercept at the stand level). Subsequently, interannual variation in growth is positively related to interannual variation in GPP and negatively related to interannual variation in drought code. Therefore, relationships based on average age (Figure 2) and on interannual variation (Table 2) do not need to be identical.

In contrast to the long-term average, the interannual variation in radial growth at any site is well explained by the photosynthetic productivity (Figure 5C) and drought index (Figure 5B). Our modeling approach described the variation of annual radial growth of trees from 1940 to 2010 (Figure 5A). Only in the 1930s does the model perform poorly. Extensive, multi-year drought events were observed in the Great Plains of Alberta in the 1930s, 1980s and early twenty first century (Marchildon et al., 2008); perhaps this drought exceeded the limits of our model. Previously reported extreme and prolonged drought events are visible both from our measured and modeled tree ring indices (Figure 5A) as radial growth was reduced during drought events. It is well known that growth is generally more sensitive to drought than photosynthetic production (e.g., Hsiao and Acevedo, 1974; McDowell, 2011; Tardieu et al., 2011). Cell and tissue expansion is very sensitive to drought as they are strongly driven by turgor pressure (Hsiao et al., 1976; Hölttä et al., 2010; Tardieu et al., 2011; Pantin et al., 2012). Drought is also known to cause an increased proportion of carbon allocation to root growth (Kozlowski and Pallardy, 2002), which was not measured in this study.

An increase in random effects of photosynthetic productivity (RE_GPP) as well as the intercept of mixed model of radial growth with relative height were observed (Figures 6A,C) but surprisingly there was no relationship between the random effects of drought code (RE_DC) and tree relative height (Figure 6B). As can be seen from Figure 6A and from the mixed model equation (cf. section Modeling Approach), the results can be interpreted as that dominant trees are more sensitive to interannual variations in GPP while interannual variations in drought affect the radial growth of suppressed and dominant trees in a similar way. A possible explanation for this phenomenon is that competition for light, which would be higher under enhanced GPP, is generally asymmetrical while competition for water, which is higher when the drought code is high, is symmetrical (Brand and Magnussen, 1988; Weiner, 1990; Burkhart and Tomé, 2012). The study on the effects of competition on radial growth of 16 common tree species in France by Kunstler et al. (2011) found that the importance of competition was greater at sites with higher heat-sums and adequate water but this effect differed between shade-tolerant and shade-intolerant tree species. For shade-tolerant species, competition only became important at high crowding indices. Another study by Prior and Bowman (2014) in temperate mesic eucalypt forests inferred that the effects of competition on tree growth was negligible in low productivity sites but had negative effects in most productive sites. As our study only considered radial growth, we cannot exclude the possibility that impacts of drought and competition index interact regarding the other growth components. Schiestl-Aalto et al. (2015) found that allocation of photosynthates between height growth and radial growth was not synchronized. Trouvé et al. (2015) found in sessile oak (*Quercus petraea* Liebl.) that the allocation between radial and height growth was affected by drought, with suppressed trees reducing their relative height growth more than dominants.

The mechanisms that mediate the asymmetry caused by shading include direct effect of shading on growth through photosynthesis, and an indirect effect through carbon allocation. Light comes from above and is shaded downward, therefore the light capture per unit foliage is larger in taller trees and consequently, growth is higher. Therefore, shorter trees with smaller crown ratios (suppressed trees) have shown reduced growth. On the other hand, symmetric competition implies that competitive effects of larger and smaller individuals are proportional to their size (Weiner, 1990), and the main effects of it seem to occur through an overall limitation of resources rather than suppression of individuals. To what extent soil resources are uniformly available to all trees remains unresolved though Weiner et al. (1997) reported availability of soil resources are uniform to all fine roots. In our case drought did not show any significant effect on the relative height of trees. It could also be that the competitive edge of taller trees is masked by their higher vulnerability to drought in relation to shorter trees (Bennett et al., 2015). Taller trees tend to be more vulnerable to drought as they generally have a lower leaf area-specific hydraulic conductance due to the longer water transport distance from roots to leaves (McDowell and Allen, 2015).

## Conclusions

Using a mixed modeling approach, and a stand level photosynthetic production model with the climate, tree-ring and competition data from mixed-species stands in Alberta, we estimated the combined effects of photosynthetic productivity, drought stress and competition on the radial growth of white spruce. In general, the effects of drought, photosynthetic productivity and social status of trees differed remarkably. While the radial growth of trees was mostly constrained by competition with minor effects from drought, a co-limitation of drought and photosynthetic productivity for radial growth exists in our stands. The interannual variations (1930–2010) of tree growth were explained by the GPP and drought index, and our modeling approach effectively described the interannual variations of growth except for the 1930s, possibly due to an exceptionally severe drought. While dominant trees are more sensitive to interannual variations of GPP, interannual variations of drought have similar effects on the radial growth of suppressed and dominant trees. Our study also demonstrated that competitive asymmetry became more pronounced when climatic conditions increased photosynthetic productivity but remained symmetric under drought. Though climate retains its fingerprint on interannual growth, these findings illustrate that intrinsic stand competitive processes remain the larger consideration in the management of white spruce in western Canadian boreal mixedwoods.

## Author contributions

FB, SA, TH, and J-GH planned and designed the research. J-GH and AD involved in field data collection. SA and FB analyzed the data and wrote the manuscript. KS, PC, J-GH, and TV involved in fund raising. GG-I, KS, TA, TH, TV, AM, PC, and J-GH commented on the manuscript.

### Conflict of interest statement

The authors declare that the research was conducted in the absence of any commercial or financial relationships that could be construed as a potential conflict of interest. The reviewer HS and handling Editor declared their shared affiliation.
